# Fracture risk prediction in postmenopausal women from GO Study: the comparison between FRAX, Garvan, and POL-RISK algorithms

**DOI:** 10.1007/s11657-024-01392-5

**Published:** 2024-05-16

**Authors:** W. Pluskiewicz, A. Werner, M. Bach, P. Adamczyk, B. Drozdzowska

**Affiliations:** 1 Department and Clinic of Internal Diseases, Diabetology, and Nephrology, Metabolic Bone Diseases Unit, Faculty of Medical Sciences in Zabrze, Medical University of Silesia, 3-Maja 13/15 Street, 41-800 Katowice, Poland; 2 Department of Applied Informatics, Silesian University of Technology, 44-100 Gliwice, Poland; 3 Department of Pediatrics, Faculty of Medical Sciences in Katowice, Medical University of Silesia, Katowice, Poland; 4 Department of Pathomorphology, Faculty of Medical Sciences in Zabrze, Medical University of Silesia, Katowice, Poland

**Keywords:** Fracture prediction, Garvan, FRAX, Osteoporosis, POL-RISK, Women

## Abstract

**Summary:**

In the longitudinal, retrospective study, the ability of the FRAX, Garvan, and POL-RISK algorithms to predict osteoporotic fractures was compared in a group of 457 women. Using the rigid threshold of 10% showed a significant discrepancy in sensitivity and specificity of all tools. New thresholds for high risk of fractures were established for each calculator separately: 6.3% for FRAX major fracture, 20.0% for Garvan any fracture, and 18.0% for POL-RISK any fracture. Such thresholds allow for improving the diagnostic accuracy of all three calculators.

**Introduction:**

The aim of the longitudinal, retrospective study was to compare three tools designed to assess fracture risk: FRAX, Garvan, and POL-RISK in their prediction of fracture incidence.

**Material:**

The study group consisted of 457 postmenopausal women with a mean age of 64.21 ± 5.94 years from the Gliwice Osteoporosis (GO) Study. Comprehensive data on clinical factors related to fractures were collected for all participants. Bone densitometry was performed at the proximal femur using the Prodigy device (GE, USA). Fracture risk was established using the FRAX, Garvan, and POL-RISK algorithms. Data on the incidence of osteoporotic fractures were collected over the last 10 years.

**Results:**

During the period of observation 72, osteoporotic fractures occurred in 63 subjects. For a preliminary comparison of the predictive value of analyzed diagnostic tools, the fracture risk threshold of 10% was used. For FRAX, the fracture probability exceeding 10% was observed only in 11 subjects who experienced fractures; thus, the fracture was properly predicted only in 22.9% of women. For Garvan, the respective value was 90.5%, and for POL-RISK, it was 98.4%. That gave a very low true positive value for FRAX and a very high false positive value for Garvan and POL-RISK. Based on ROC curves, new thresholds for high risk of fractures were established for each calculator separately: 6.3% for FRAX major fracture, 20.0% for Garvan any fracture, and 18.0% for POL-RISK any fracture. Such thresholds improve the diagnostic accuracy of all compared fracture prediction tools.

**Conclusion:**

The current study showed that different fracture risk assessment tools, although having similar clinical purposes, require different cut-off thresholds for making therapeutic decisions. Better identification of patients requiring therapy based on such an approach may help reduce the number of new fractures.

## Introduction

The most important point in regard to osteoporotic subjects is fracture prediction. Osteoporosis commonly has no clear clinical symptoms, and thus, it is called a “silent bone thief.” A large number of patients indicate that osteoporosis is an epidemic in modern societies. Osteoporotic fractures are the essential symptom of bone loss and they are usually the consequence of falls from a standing height. Fractures may occur in several skeletal sites, and the most common are fractures of the hip, spine, forearm, and arm. Fractures in those locations are called “major osteoporotic fractures.” Prior fragility fracture is an important risk factor for subsequent fracture [1]. Therefore, the primary aim in patient management is to avoid the first fracture. Usually, osteoporosis is not recognized at early stages of the disease, and the most important point is the possibility to establish fracture risk and predict future ones. In recent years some methods designed to assess fracture risk have been developed. There are FRAX [2], Garvan [3, 4], and POL-RISK [5, 6] among them. Garvan and POL-RISK allow for the estimation of fracture risk, whereas FRAX enables the establishment of fracture probability limited by life expectancy. For practitioners, the measurements of fracture risk are helpful in the identification of subjects requiring pharmacologic therapy. Some studies presented the results of fracture prediction in various populations [7–9]. The early assessment of fracture risk is an important point for adequate daily patient management in order to avoid the first fracture. Several authors discussed the problem of fracture risk assessment and prediction [10–12]. In other studies, the comparisons between the FRAX and Garvan methods were presented [13–23]. Such data on fracture prediction are helpful for practitioners in daily work with patients.

The aim of the current study was the comparison of fracture prediction established by FRAX, Garvan, and POL-RISK in presented earlier cohort of the GO Study [24, 25]. The secondary goal was to establish an optimal threshold of fracture risk for initiation of pharmacologic therapy.

## Material

The study group comprised a cohort of postmenopausal women from the GO Study [24, 25]. Briefly, the study sample consisted of postmenopausal women recruited in the Outpatient Osteoporotic Clinic in South Poland (city Gliwice, Upper Silesia). Data on clinical risk factors for osteoporosis and fractures were collected in all subjects. The skeletal status was assessed at the femoral neck using the Prodigy device (GE, USA). The clinical characteristics of the studied subjects are presented in Table 1. The information on fracture incidence for a period of 10 years was gathered. Either the review of patients’ charts or phone calls allowed us to identify individuals who experienced the fracture during the previous 10 years. All data were collected by one investigator (WP).

**Table 1:** Clinical characteristics of the whole study group and subgroups with and without fracture in follow-up and the results of baseline fracture risk assessment

Variable	Whole group, *n* = 457	Fractured patients, *n* = 63	Non-fractured patients, *n* = 394	Major fractures patients, *n* = 48
Age (years)	64.21 ± 5.94	65.92 ± 5.61*	63.94 ± 5.95	66.35 ± 5.75*
Body mass (kg)	71.25 ± 13.3	70.19 ± 13.63	71.41 ± 13.25	71.7 ± 14.3
Height (cm)	158.75 ± 5.97	158.38 ± 5.56	158.8 ± 6.04	158.6 ± 5.62
BMI (kg/m^2^)	28.25 ± 5.31	27.91 ± 5.82	28.32 ± 5.22	28.23 ± 6.11
T-score for FN BMD	− 1.5 ± 0.87	− 1.86 ± 0.85*	− 1.44 ± 0.86	− 1.84 ± 0.90*
FRAX major fracture	5.82 ± 3.77	8.47 ± 6.53	5.40 ± 2.91	8.17 ± 5.27
Garvan any fracture	19.17 ± 13.97	29.52 ± 20.06	17.52 ± 11.96	31.52 ± 20.75
POL-RISK any fracture	19.86 ± 9.81	26.45 ± 13.84	18.80 ± 8.56	27.83 ± 14.45

^*^Mean age and T-score significantly different in all fractured subgroups than in non-fractured patients

During the 10-year period of observation, 72 osteoporotic fractures occurred in 63 subjects. These fractures were located in the following skeletal sites: forearm — 20, spine — 17, hip — 13, ankle 9, arm — 7, and other 6. Single fractures were recorded in 56 women, whereas 7 women reported multiple (2 or 3) fractures.

## Methods

Baseline fracture risk was established using the FRAX, Garvan, and POL-RISK algorithms. Obviously, for FRAX, the fracture risk was expressed as fracture probability limited by life expectancy.

Because POL-RISK does not measure the risk of hip fracture, only any fracture prediction (Garvan, POL-RISK) and major fractures (FRAX) were presented.

For a preliminary comparison of the predictive value of the analyzed diagnostic tools, the fracture risk threshold of 10% was used, according to local recommendations [26].

Proximal femur bone densitometry was performed using the Prodigy device (GE Lunar). Based on repeated measurements the precision (CV%) of DXA measurements at FN was established at 1.6%. Calculations of fracture risk were performed based on femoral neck (FN) BMD.

All measurements were done by one experienced DXA technician.

## Statistics

All statistical calculations were performed with the use of Statistica 13.3 software (StatSoft, Tulsa, OK, USA) and PQStat v.1.8.2.238 (PQStat Software, Plewiska, Poland; https://pqstat.pl). The mean values and standard deviations were used for descriptive statistics of continuous variables. The prevalence of qualitative features was presented as the number of subjects with percentage values. Values of sensitivity, specificity, and balanced accuracy (BAcc) were used to compare the predictive powers of compared fracture risk algorithms. BAcc was chosen instead of accuracy (Acc), as the analyzed dataset was characterized by a high degree of imbalance. In medical decision-making, data is usually highly imbalanced, i.e., high-risk patients are in the minority, whereas correct prediction of their disease is critical. It means that bad recognition of the minority subjects has much more serious consequences for the patients and medics than the creation of a so-called “false alarm” when the low-risk subjects of the majority class are assigned to the minority one [27]. Therefore, the conventional accuracy, calculated as a percentage of the examples correctly predicted by the method, is not only skewed by the imbalance but also inappropriate. Hence, to assess the quality of the analyzed tools, balanced accuracy (BAcc), which is the average of sensitivity and specificity, was applied in the study. To verify the prediction accuracy of the analyzed diagnostic tools, the receiver operating characteristic (ROC) was studied, as well as the area under the curve (AUC) was calculated using the DeLong method. The alternative cut-offs determining high or low fracture risk were established based on ROC curves with the “distance from the top left corner” method. Based on logistic regression analysis, calibration plots for all three analyzed calculators at both compared cut-off thresholds — the standard one and the one determined by ROC analysis were prepared.

A *p*-value at a level of 0.05 was regarded as statistically significant.

The statistical power of the test was determined post hoc for the assumed *α* (level of significance) at 0.05, the sample size of 457 subjects (divided into two subgroups of 63 and 394) and for the effect size between subgroups 0.8 (calculated based on Cohen’s method) was 0.99.

## Results

Table 1 presents clinical characteristics, FN T-score values, and fracture risk calculated for major (FRAX) or any (Garvan and POL-RISK) fractures in all patients and subgroups.

As one might expect, age was significantly higher, and the T-score was significantly lower for women in each fractured subgroups (*p* < 0.05). To compare the predictive value of analyzed calculators in relation to fractures observed during the 10-year observation, all the subjects were first categorized into low or high fracture probability/risk of major/any fracture according to thresholds of 10%. Table 2 presents information on the number of subjects classified in high-risk category, and the number of observed fractures during the follow-up period.

**Table 2:** The number of subjects classified in the high-risk category (fracture risk/probability > 10%) and the number of fractures observed during the follow-up period

Fracture prediction tool	Subjects at high risk (predicted fractures)	Observed fractures (total)*	Fractures in high-risk subgroup (predicted and observed)**	Fractures in low-risk subgroup (unpredicted but observed)**
FRAX major fracture	40	48	11 (22.9%)	37 (77.1%)
Garvan any fracture	372	63	57 (90.5%)	6 (9,5%)
POL-RISK any fracture	430	63	62 (98.4%)	1 (1.6%)

^*^The number of the observed fractures is given as major fractures for FRAX and all (any) fractures for Garvan and POL-RISK

^**^The percentage values are given in relation to the total number of fractures observed in each category

For FRAX, the fracture probability exceeding 10% was observed only in 11 subjects with the observed fractures, and thus the fracture was properly predicted only in 22.9% of women with major fractures (11 patients out of 48 ones). For Garvan, the respective value was 90.5% (57 patients out of 63 with any fracture), and for POL-RISK it was 98.4% (62 patients out of 63 with any fracture). Therefore, FRAX showed significantly lower true positive (TP) results in comparison to Garvan and POL-RISK. It means that only 22.9% of patients who should receive therapy would be properly identified to start the treatment based on the FRAX method. Based on the Garvan algorithm, only 9.5% (6 of 63 fractured patients) would not be recommended to start treatment, and in the case of POL-RISK, only one patient (1.6% of fractured ones) would miss the treatment.

On the other hand, the Garvan and POL-RISK calculators showed a much higher prediction of fractures than actually observed, which means those algorithms provide a high ratio of false positive (FP) results. According to Garvan, 315 patients, and according to POL-RISK, even 368 patients were identified as high-risk subjects but did not experience fracture during the follow-up period. One can say that those patients would receive unnecessary treatment. For FRAX, the number of such patients was clearly lower — only 29. In Table 3, there are given data for sensitivity, specificity, and balanced accuracy (BAcc) for three methods.

**Table 3:** Sensitivity, specificity (with 95% CI), and balanced accuracy (BAcc) for FRAX, Garvan, and POL-RISK calculated for “standard” cut-off of 10%

Method	Sensitivity	Specificity	BAcc
FRAX major fracture	0.2292 (0.1251–0.3767)	0.929 (0.8986–0.9512)	0.5791
Garvan any fracture	0.9048 (0.7976–0.9607)	0.2005 (0.1628–0.2442)	0.5526
POL-RISK any fracture	0.9841 (0.9032–0.9992)	0.066 (0.0444–0.0964)	0.5251

**Fig. 1:**
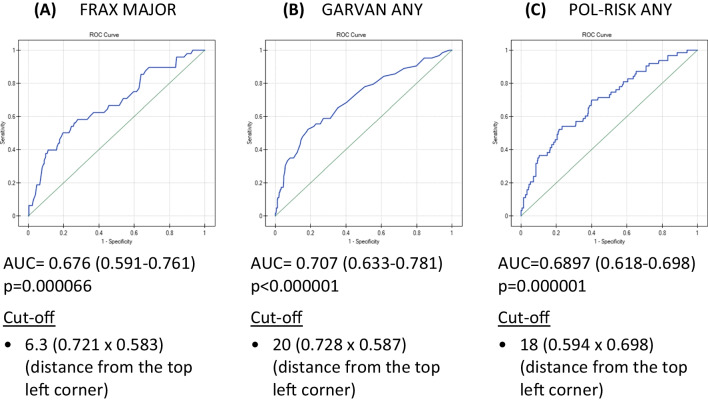
Analysis of receiver operator characteristic (ROC) curves for FRAX, Garvan, and POL-RISK; null hypothesis: true area = 0.5; the numbers in parentheses indicate specificity and sensitivity values for the proposed cut-off

Such a significant discrepancy between sensitivity and specificity clearly shows that using the “standard” cut-off at 10% does not allow achieving the optimal predictive value of compared tools. According to [19], an acceptable model should have both specificity and sensitivity of 50–79%. The ROC analyses were also performed to establish the separate cut-offs for each calculator, improving the accuracy of fracture prediction. The achieved ROC curves for each diagnostic tool are presented in Fig. 1.

When determining the cut-off thresholds, the method of measuring the distance from the top left corner was used. The following fracture risk threshold values were obtained, corresponding to the balanced values of sensitivity and specificity, giving optimal diagnostic accuracy: FRAX major fracture — 6.3%, Garvan any fracture — 20.0%, and POL-RISK any fracture — 18.0%. Table 4 shows the sensitivity, specificity, and balanced accuracy (BAcc) values after applying the given cut-offs. It can be seen that the BAcc value increased compared to the values obtained for previously tested thresholds (Table 3) for all tested diagnostic tools.

**Table 4:** Sensitivity, specificity (with 95% CI), and balanced accuracy (BAcc) for FRAX, Garvan, and POL-RISK calculated for the newly determined cut-offs

Method	Cut-off	Sensitivity	Specificity	BAcc
FRAX major fracture	6.3%	0.5833 (0.4328–0.7207)	0.7213 (0.6746–0.7637)	0.6523
Garvan any fracture	20%	0.5873 (0.4564–0.7076)	0.7284 (0.6811–0.7712)	0.6579
POL-RISK any fracture	18%	0.6984 (0.5682–0.8043)	0.5939 (0.5435–0.6425)	0.6462

Hence, the presented analyses confirm the validity of using different decision thresholds for all compared calculators.

The relationship between observed fractures and the estimated number of subjects predicted to have fractures based on the “new” cut-offs is presented in Table 5.

**Table 5:** The number of subjects classified in the high-risk category with respect to the newly determined cut-off values and the number of observed fractures during the follow-up period

Method	Cut-off	Subjects at high risk (predicted fractures)	Observed fractures (total)*	Fractures in high-risk subgroup (predicted and observed)**	Fractures in low-risk subgroup (unpredicted but observed)**
FRAX major fracture	6.3%	142	48	28 (58.3%)	20 (41.7%)
Garvan any fracture	20%	144	63	37 (58.7%)	26 (41.3%)
POL-RISK any fracture	18%	204	63	44 (69.8%)	19 (30.2%)

^*^The number of the observed fractures is given as major fractures for FRAX and all (any) fractures for Garvan and POL-RISK

^**^The percentage values are given in relation to the total number of fractures observed in each category

In comparison to data from Table 2, a clear increase in properly predicted fractures by FRAX is achieved. In the case of the Garvan method and the POL-RISK algorithm, there is a significant reduction in the number of high-risk subjects with an acceptable decrease in the level of properly predicted fractures (similar to the prediction achieved for the FRAX algorithm).

**Fig. 2:**
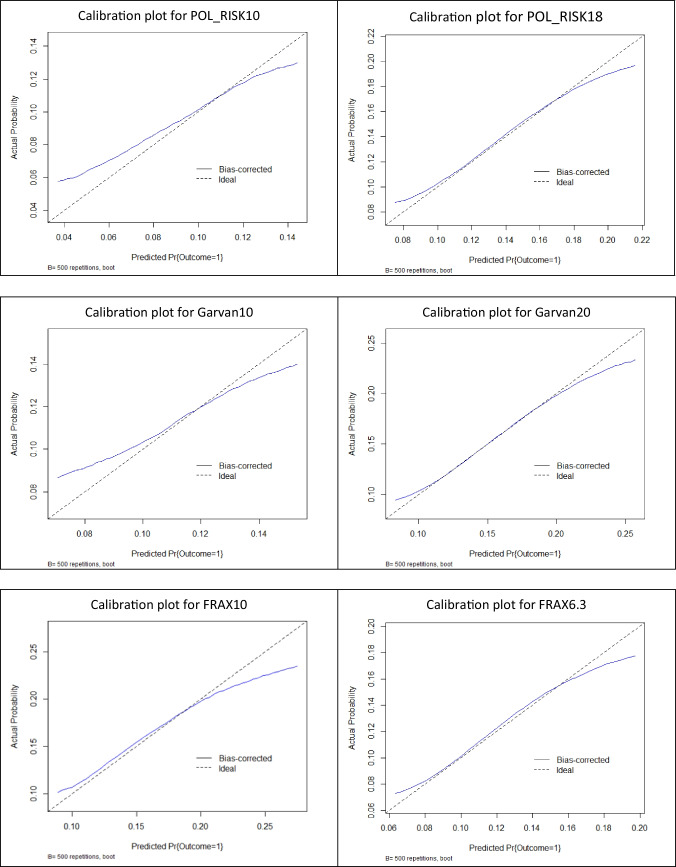
Calibration plots (based on logistic regression analysis) for fracture prediction in analyzed calculators at both compared cut-off thresholds — the standard one and the one determined in ROC analysis

Finally, to support the comprehensive presentation of differences in fracture risk assessment provided by standard and newly determined cut-off thresholds, calibration plots based on logistic regression analysis for all three analyzed calculators were prepared. They are presented in Fig. 2. It can be noticed that for all diagnostic tools, the prediction curves are much closer to the “ideal line” in the case of thresholds calculated in the current study by ROC analyses in comparison to “standard” cut-off values. This additionally supports the validity of determining cut-off thresholds in the local population rather than the universal use of standard thresholds.

## Discussion

The problem of accurate fracture prediction is essential in osteoporotic patients. Usually, osteoporosis has no clear clinical signs of the disease, and the first symptom is a fracture of typical low-trauma origin. The first fracture significantly increases the risk of subsequent fracture; therefore, the therapy should be initiated earlier. Due to an enormous number of individuals with osteoporosis, objective medical as well as economic aspects do not allow recommending the initiation of therapy for all patients. For practitioners, the most important point is to identify subjects at high risk. Methods designed to establish fracture risk (or probability) potentially should be helpful. In the current study, we presented the results of fracture prediction established by three different methods. Significant differences were noted in regard to the identification of subjects at high fracture risk, e.g., those who should be treated because of incident fracture. For the threshold of 10%, only 23% patients were classified as those who require therapy by FRAX, while for POL-RISK, and Garvan, the majority of patients would be treated (98% and 90%, respectively). This observation indicates that using FRAX, the majority of high-risk patients would not be identified, and therapy would not be started. However, good values of TP for POL-RISK and Garvan were accompanied by a great number of subjects with FP classification. Therefore, many patients would be treated unnecessarily. Presented results suggested that the new threshold of fracture risk should be proposed in order to recommend treatment in the majority of high-risk patients. The second important point is to avoid treatment in patients at low risk. We consider that the threshold of 18% established in the current study for the POL-RISK calculator might be used in daily practice.

This threshold is the most important finding of the study. With acceptable sensitivity, specificity, and accuracy, the therapy may be started in high-risk patients, and the number of patients unnecessarily treated is much lower.

One should also take into consideration that each recommendation should be based not only on *pure* medical points but also economic aspects must be added. The general cost for the health system, including all points (medication reimbursement, surgery, rehabilitation, and many others) should allow for establishing a threshold of high risk for use in daily practice. Such considerations are pointed out by the authors of the Garvan algorithm (www.fractureriskcalculator.com). For example, according to these recommendations, any fracture risk assessment values exceeding 26% allow using reimbursement medications, and those below 14% do not. The patients classified in the range between 14 and 26% should be assessed individually. One may consider that the *pure* medical threshold is 14%, and the *economic* threshold is 26%. Our threshold of 18% for POL-RISK should be treated as medical one, and further analysis in order to reveal a threshold fitted to economic aspects is necessary. In the near future, we plan to perform the study in order to establish the optimal fracture risk threshold for use in daily practice.

Irrespective of the threshold used (either 10 or 18%) some patients would not be adequately identified. Some will not be treated, and the other ones will be unnecessarily treated. Such observations suggest that it is not possible to create a tool that would replace physician thinking, and individualization of the initiation of treatment will always be necessary.

A similar need to differentiate the threshold for low and high risk of future fractures was also demonstrated in a recently published study based on analyses in an epidemiological sample of postmenopausal women from the RAC-OST-POL Study [28]. In that study, the optimal threshold for prediction of major fractures using FRAX was 6.0%, which is very close to the value obtained in current analyses (6.3%). The optimal threshold for any fracture in the Garvan algorithm established in the RAC-OST-POL cohort was 14.4%, and this value is lower than calculated in the current study. The difference may be explained by the specific character of both study cohorts. The RAC-OST-POL sample is population-representative and, therefore, also includes healthy people. The currently presented group was recruited in an osteoporosis outpatient clinic, which may result in a smaller proportion of healthy people or those with risk factors present only to a minimal extent. POL-RISK algorithm was not taken into consideration in the cited study.

The results presented by other authors should be discussed. In the study by Donaldson et al. [7], the authors compared various methods in order to classify patients as candidates for treatment. Always some patients at high risk were missed, and in others, unnecessary treatment would be offered. An interesting comparison between the utility of absolute risk prediction using the FRAX and Garvan methods was presented in the next study [16]. Only 8.9% of women who sustained a fracture had an estimated fracture risk ≥ 20% using FRAX compared with 53.3% using Garvan. The authors compared AUC, sensitivity, specificity, FP, FN, and accuracy for thresholds 10, 20, and 30%. Always the accuracy had the highest value for threshold 30% but the exact threshold recommended for daily use was not given. Other authors compared FRAX and Garvan in a great cohort of women from the Women’s Health Initiative Study [18]. The final conclusion was that there is no useful threshold for either methods. In the Belgian study with the use of the threshold of 20%, only a small part of high-risk patients would be treated according to results given by FRAX and Garvan, and a slightly better classification was performed by Garvan [20]. Overall, data given by other authors suggest that there is no simple tool that can accurately identify a high-risk person and at the same time recommend treatment. Always in some patients, the fracture risk is either over- or underestimated. In our opinion, the numerous studies on the use of fracture risk assessment tools should be completed with clear recommendations for practitioners. Long variables expressing available methods like AUC, accuracy, sensitivity, or specificity are not sufficiently helpful in daily practice with patients. We believe that the threshold of fracture risk for treatment initiation is an essential condition for adequate patients’ management. Indicating a certain threshold can be very helpful, but one should not forget about an individual approach to the patient. The established threshold should be treated as a guideline rather than an immutable value.

The study has some limitations. We observed only women, the sample size of patients and number of fractures might be greater. POL-RISK does not establish separate risk for hip fractures and only any fracture risk was established. One should also remember that the group studied was not an epidemiological sample.

The current study showed that different fracture risk assessment tools, although having similar clinical purposes, require different cut-off thresholds for making therapeutic decisions. Adjusting such thresholds separately for each calculator improves their diagnostic sensitivity and specificity. Better identification of patients requiring therapy based on such an approach may help reduce the number of new fractures.

## References

[1] Klotzbuecher CM, Ross PD, Landsman PD, Abbot PA, Berger M (2000) Patients with prior fractures have increased risk of future fracture: a summary of the literature and statistical synthesis. J Bone Miner Res 15:721–727 10780864 10.1359/jbmr.2000.15.4.721

[2] Kanis JA, Johnell O, Oden A et al (2008) FRAX™ and the assessment of fracture probability in men and women from the UK. Osteoporos Int 19:385–397 18292978 10.1007/s00198-007-0543-5 PMC2267485

[3] Nguyen ND, Frost SA, Center JR, Eisman JA, Nguyen TV (2007) Development of a nomogram for individualizing hip fracture risk in men and women. Osteoporos Int 18:1109–1117 17370100 10.1007/s00198-007-0362-8

[4] Nguyen ND, Frost SA, Center JR, Eisman JA, Nguyen TV (2008) Development of prognostic nomograms for individualizing 5-year and 10-year fracture risks. Osteoporos Int 19:1431–1434 18324342 10.1007/s00198-008-0588-0

[5] Adamczyk P, Werner A, Bach M et al (2018) Risk factors for fractures identified in the algorithm developed in 5-year follow-up of postmenopausal women from RAC-OST-POL Study. J Clin Densitom 21(2):213–219 28826886 10.1016/j.jocd.2017.07.005

[6] Pluskiewicz W, Adamczyk P, Werner A, Bach M, Drozdzowska B. (2023) POL-RISK: an algorithm for 10-year fracture risk prediction in the postmenopausal women from the RAC-OST-POL Study. Pol Arch Intern Med 133:16395. 10.20452/pamw.16395 10.20452/pamw.16395 36601872

[7] Donaldson MG, Cawthon PM, Schousboe JT et al (2011) Novel methods to evaluate risk models. J Bone Miner Res 26:1767–1773 21351143 10.1002/jbmr.371 PMC3544194

[8] Rubin KH, Friis-Holmberg T, Hermann AP, Abrahamsen B, Brixen K (2013) Risk assessment tools to identify women with increased risk of osteoporotic fracture: complexity or simplicity? A systematic review. J Bone Miner Res 28:1701–1717 23592255 10.1002/jbmr.1956

[9] Beaudoin C, Moore L, Gagne M et al (2019) Performance of predictive tools to identify individuals at risk of non-traumatic fracture: a systematic review, meta-analysis and meta-regression. Osteoporos Int 30:721–740 30877348 10.1007/s00198-019-04919-6

[10] Chen SY, Chen YJ, Cheng CH, Hwang HF, Chen CY, Lin MR (2016) Comparison of different screening tools for identifying fracture/osteoporosis risk among community-dwelling older people. Medicine 95(20):e3415 27196447 10.1097/MD.0000000000003415 PMC4902389

[11] Nguyen TV, Eisman JA (2017) Fracture risk assessment: from population to individual. J Clin Densitom 20:368–378 28729045 10.1016/j.jocd.2017.06.023

[12] Nguyen TV (2018) Individualized fracture risk assessment: state-of-the-art and room for improvement. Osteoporosis and Sarcopenia 4:2–10 30775534 10.1016/j.afos.2018.03.001 PMC6362956

[13] Pluskiewicz W, Adamczyk P, Franek E, Leszczynski P, Sewerynk E, Wichrowska H et al (2010) Ten-year probability of osteoporotic fracture in 2012 Polish women assessed by FRAX and nomogram by Nguyen et al. – conformity between methods and their clinical utility. Bone 46:1661–7 20156606 10.1016/j.bone.2010.02.012

[14] Pluskiewicz W, Adamczyk P, Franek E et al (2014) FRAX calculator and Garvan nomogram in male osteoporotic population. Aging Male 17:174–182 24456527 10.3109/13685538.2013.875991

[15] Bolland MJ, Siu AT, Manson BH et al (2011) Evaluation of the FRAX and Garvan fracture risk calculators in older women. J Bone Miner Res 26:420–427 20721930 10.1002/jbmr.215

[16] van Geel TACM, Eisman JA, Geusens PP, van den Bergh JPW, Center JR, Dinant GJ (2014) The utility of absolute risk prediction using FRAX and Garvan fracture risk calculator in daily practice. Maturitas 77:174–179 24287178 10.1016/j.maturitas.2013.10.021

[17] Billington EO, Gamble GD, Reid IR (2016) Reasons for discrepancy in hip fracture risk estimates using FRAX and Garvan calculators. Maturitas 85:11–18 26857874 10.1016/j.maturitas.2015.12.003

[18] Crandall CJ, Larson J, LaCroix A et al (2018) Predicting fracture risk in younger postmenopausal women: comparison of the Garvan and FRAX risk calculators in the women’s Health Initiative Study. J Gen Intern Med 34:235–242 30334182 10.1007/s11606-018-4696-z PMC6374270

[19] Holloway-Kew KL, Zhang Y, Betson AG et al (2019) How well do FRAX (Australia) and Garvan calculators predict incident fractures? Data from the Geelong Osteoporosis Study. Osteoporos Int 30:2129–2139 31317250 10.1007/s00198-019-05088-2

[20] Baleanu F, Iconaru L, Charles A et al (2021) Indepedent external validation of FRAX and Garvan fracture risk calculators: a sub-study of the FRISBEE Cohort. JBMR Plus (WOA) 5(9):e10532 10.1002/jbm4.10532 PMC8441269 34532617

[21] Dagan N, Cohen-Stavi Ch, Leventer-Roberts M, Balicer RD (2017) External validation and comparison of three prediction tools for risk of osteoporotic fractures using data from population based electronic health records: retrospective cohort study. BMJ 356:i6755. 10.1136/bmj.i6755 28104610 10.1136/bmj.i6755 PMC5244817

[22] Todorow G, Brook S, Quah Qin Xian N, Von Widekind S, Freudenthal B, Comninos AN (2022) Comparison of fracture risk calculators in elderly fallers: a hospital-based cross-sectional study. BMJ Open 12:e060282. 10.1136/bmjopen-2021-060282 10.1136/bmjopen-2021-060282 PMC9274535 35820750

[23] Iconaru L, Charles A, Baleanu F et al (2022) Prediction model of an imminent fracture after an index fracture – models derived from the Frisbee Cohort. J Bone Miner Res 37:59–67 34490908 10.1002/jbmr.4432

[24] Pluskiewicz W, Adamczyk P, Drozdzowska B (2021) The significance of height loss in postmenopausal women. The results from GO Study. Int J Clin Pract 75:14009. 10.1111/ijcp.14009 10.1111/ijcp.14009 33411978

[25] Pluskiewicz W, Adamczyk P, Drozdzowska B (2021) Height loss in postmenopausal women-do we need more for fracture risk assessment? Results from the GO Study. Osteoporos Int 32:2043–2049 33818635 10.1007/s00198-021-05941-3 PMC8510894

[26] Lorenc R, Głuszko P, Franek E et al (2017) Recommendations for diagnostic and therapeutic management in osteoporosis in Poland– update [in Polish]. Endokrynol Pol 68:1–1829168544

[27] Werner A, Bach M, Pluskiewicz W (2016) The study of preprocessing methods’ utility in analysis of multidimensional and highly imbalanced medical data. In: Rostański M, Pikiewicz P, Buchwald P, Maczka K (eds) Proceedings of the 11th Scientific Conference IIIS, Scientific publisher of WSB University in Dąbrowa Górnicza

[28] Pluskiewicz W, Werner A, Bach M, Adamczyk P, Drozdzowska B (2023) Optimal fracture prediction thresholds for therapy onset, established from FRAX and Garvan algorithms: a longitudinal observation of the population representative female cohort from the RAC-OST-POL Study. Arch Osteoporos 18(1):136 37973685 10.1007/s11657-023-01346-3 PMC10654207

